# Plasma-Activated Water Triggers Rapid and Sustained Cytosolic Ca^2+^ Elevations in *Arabidopsis thaliana*

**DOI:** 10.3390/plants10112516

**Published:** 2021-11-19

**Authors:** Enrico Cortese, Alessio G. Settimi, Silvia Pettenuzzo, Luca Cappellin, Alessandro Galenda, Alessia Famengo, Manuele Dabalà, Vanni Antoni, Lorella Navazio

**Affiliations:** 1 Department of Biology, University of Padova, Via U. Bassi 58/B, 35131 Padova, Italy; enrico.cortese@unipd.it; 2 Department of Industrial Engineering, University of Padova, Via F. Marzolo 9, 35131 Padova, Italy; alessiogiorgio.settimi@unipd.it (A.G.S.); manuele.dabala@unipd.it (M.D.); 3 Center Agriculture Food Environment (C3A), University of Trento, Via E. Mach 1, 38010 San Michele all’Adige, Italy; silvia.pettenuzzo-1@unitn.it; 4 Research and Innovation Centre, Edmund Mach Foundation, Via E. Mach 1, 38010 San Michele all’Adige, Italy; 5 Department of Chemical Sciences, University of Padova, Via F. Marzolo 1, 35131 Padova, Italy; luca.cappellin@unipd.it; 6 CNR Institute of Condensed Matter Chemistry and Technologies for Energy (ICMATE), Corso Stati Uniti 4, 35127 Padova, Italy; alessandro.galenda@cnr.it (A.G.); alessia.famengo@cnr.it (A.F.); 7 Consorzio RFX, Corso Stati Uniti 4, 35127 Padova, Italy; vanni.antoni@igi.cnr.it; 8 Botanical Garden, University of Padova, Via Orto Botanico 15, 35123 Padova, Italy

**Keywords:** aequorin, *Arabidopsis thaliana*, calcium signalling, cytosolic Ca^2+^ changes, plasma-activated water, plasma torch, reactive oxygen species, reactive nitrogen species

## Abstract

Increasing evidence indicates that water activated by plasma discharge, termed as plasma-activated water (PAW), can promote plant growth and enhance plant defence responses. Nevertheless, the signalling pathways activated in plants in response to PAW are still largely unknown. In this work, we analysed the potential involvement of calcium as an intracellular messenger in the transduction of PAW by plants. To this aim, *Arabidopsis thaliana* (Arabidopsis) seedlings stably expressing the bioluminescent Ca^2+^ reporter aequorin in the cytosol were challenged with PAW generated by a plasma torch. Ca^2+^ measurement assays demonstrated the induction by PAW of rapid and sustained cytosolic Ca^2+^ elevations in Arabidopsis seedlings. The dynamics of the recorded Ca^2+^ signals were found to depend upon different parameters, such as the operational conditions of the torch, PAW storage, and dilution. The separate administration of nitrate, nitrite, and hydrogen peroxide at the same doses as those measured in the PAW did not trigger any detectable Ca^2+^ changes, suggesting that the unique mixture of different reactive chemical species contained in the PAW is responsible for the specific Ca^2+^ signatures. Unveiling the signalling mechanisms underlying plant perception of PAW may allow to finely tune its generation for applications in agriculture, with potential advantages in the perspective of a more sustainable agriculture.

## 1. Introduction

Cold atmospheric plasmas are weakly ionized gases that can be generated in ambient air. At a relatively low consumption of energy, they constitute a unique delivery system of a rich family of short- and long-lived chemicals, such as reactive oxygen (ROS) and nitrogen (RNS) species, called RONS when grouped together. Cold plasmas have already proven effective in medicine applications such as in regenerative medicine for blood coagulation and dental treatment, as well as in sanitizing surfaces and medical tools, and further applications, such as anti-cancer treatments, are under investigation [1]. When interacting with a liquid, cold plasmas can generate further new chemical species, as in the case of the so-called plasma-activated water (PAW). The nature and concentration of the RONS generated depend on the sources and gases used for plasma generation, on the chemical environment, and can be modulated by varying parameters such as voltage, distance between the liquid and the plasma, exposure time, and type of electrodes used. In the generated PAW short-living species such as hydroxyl- (^•^OH), superoxide- (O_2_^•−^), nitric oxide- (^•^NO) radicals, and ozone (O_3_) are formed and further react, yielding nitric oxide (NO), nitrate (NO_3_^−^) and nitrite (NO_2_^−^), peroxynitrite (ONOO^−^), and hydrogen peroxide (H_2_O_2_) [2].

The complex chemistry occurring during the PAW generation has recently attracted a great deal of interest due to a variety of applications in agriculture and in the food sector [2,3,4,5]. In plant biology, cold plasma and PAW have been shown to increase the seed germination rate, even under osmotic and saline stresses, as well as to promote plant growth [6,7,8,9,10,11]. Moreover, PAW irrigation of tomato plants has been reported to induce defence gene expression [12,13] and accumulation of the defence hormones salicylic acid and jasmonic acid [12,14]. A differential expression of genes involved in the main plant defence pathways was also confirmed in PAW-treated periwinkle and grapevine plants [15]. These studies suggest that PAW can play beneficial roles in agriculture by promoting plant growth and pre-alerting plant defence prior to a potential subsequent attack by pathogens, a phenomenon defined as “priming” [8,16]. PAW may, therefore, represent an attractive eco-friendly alternative to pesticides, whose administration in bulk quantities represent a matter of growing concern for their impact on the environment. Nevertheless, studies addressing the signalling pathways activated in plants in response to PAW have been lacking so far. 

In this work, we investigated the signalling mechanisms underpinning the effects played by PAW on plants. In particular, we evaluated the potential involvement of calcium in the plant perception and transduction of the mixture of molecules contained in PAW. Calcium is a universal signalling element involved in a wide range of physiological processes in all living organisms [17]. In plants, Ca^2+^ serves as an intracellular messenger of primary importance in many different signal transduction pathways [18]. A plethora of abiotic stimuli, such as touch/wind [19,20], salinity, drought [21,22,23], oxidative stress [24], and cold/heat stress [25,26,27,28,29,30,31], as well as biotic stimuli in pathogenic and beneficial plant-microbe interactions [32,33], have been shown to evoke in plants specific spatio-temporal Ca^2+^ signals, which are further transduced by Ca^2+^ sensor proteins into transcriptional and metabolic responses [34,35,36]. Notably, Ca^2+^-based signalling circuits are well conserved along the green lineages, from algae to embryophytes [30,37]. 

To test the effect of PAW on the induction of transient changes in the cytosolic concentration of the ion ([Ca^2+^]_cyt_), we used as an experimental system a transgenic line of the model plant *Arabidopsis thaliana* (Arabidopsis) stably expressing the genetically encoded Ca^2+^ indicator aequorin in the cytosol. The obtained results showed that Arabidopsis perception of PAW generated by two different plasma torches is mediated by rapid and sustained cytosolic Ca^2+^ elevations. Further studies are needed in the future to address conserved and unique features of Ca^2+^-mediated sensing mechanisms of PAW in phylogenetically distant plant species, as well as in plants of economic interest. A better understanding of the biochemical and molecular bases of plant perception of PAW may allow to finely tune the chemical composition of PAW for an optimal application in agriculture. 

## 2. Results

### 2.1. Generation of PAW by Plasma Torch 

During the course of this work two plasma torches (torch #1 and torch #2) were used, both consisting of a non-transferred arc generated through a narrow nozzle less than 1 cm wide, so that a relatively high-power plasma could be generated and concentrated in a relatively narrow surface. To produce PAW, samples of 50 mL deionized H_2_O were treated for different time intervals, ranging from 1 to 10 min, with the torch at a distance of 1 to 10 cm from the H_2_O surface, in a cooling bath of ice and salt. Operational parameters of the plasma torch were set to operate in a range of power 450–1800 W and with a pressure from 1 to 3 bar. Upon generation, PAW was quickly fractionated in small single-use aliquots and immediately frozen in liquid N_2_.

### 2.2. PAW Triggers a Cytosolic Ca^2+^ Increase in Aequorin-Expressing Arabidopsis thaliana Seedlings

**Figure 1:**
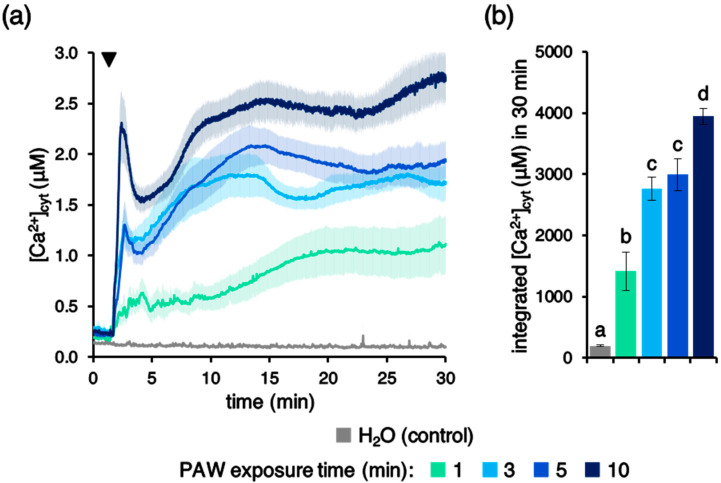
Monitoring of changes in cytosolic Ca^2+^ concentration ([Ca^2+^]_cyt_) induced by plasma-activated water (PAW) in *Arabidopsis thaliana* (Arabidopsis). Ca^2+^ measurement assays were conducted in Arabidopsis seedlings stably expressing aequorin in the cytosol. Seven-day-old intact seedlings were challenged with 1:2 dilutions of different PAWs, obtained by exposing deionized H_2_O to cold plasma (generated at 900 W power, torch #1) for different time intervals: 1 min (green), 3 min (light blue), 5 min (blue), or 10 min (dark blue). Untreated deionized H_2_O was administered to control samples (grey). (**a**) Data are the means (solid lines) ± SE (shading) of six seedlings derived from three independent growth replicates. The arrowhead indicates the time of stimulus application (at 100 s); (**b**) statistical analyses of integrated [Ca^2+^]_cyt_ dynamics over 30 min. Bars labelled with different letters differ significantly (*p* < 0.05, Student’s *t* test).

To evaluate the potential involvement of Ca^2+^ signalling in the perception of PAW by plants, an Arabidopsis line stably expressing the bioluminescent Ca^2+^ indicator aequorin in the cytosol was used [38,39,40]. Ca^2+^ measurement assays were carried out in 7-day-old transgenic Arabidopsis intact seedlings, which were challenged with PAW generated by the above-described plasma torch settings. Plant treatment with PAW induced rapid and sustained cytosolic Ca^2+^ increases (Figure 1a), whose magnitude was found to correlate with the duration of the exposure of H_2_O to plasma (Figure 1b). No [Ca^2+^]_cyt_ changes were observed in control samples, in which deionized H_2_O (without activation by plasma) was applied to seedlings (Figure 1a). These data demonstrate that PAW sensing by plants occurs through intracellular Ca^2+^ changes, characterized by a specific signature. 

### 2.3. The Dynamics of the Elicited Ca^2+^ Signals Depend on PAW Features

**Figure 2:**
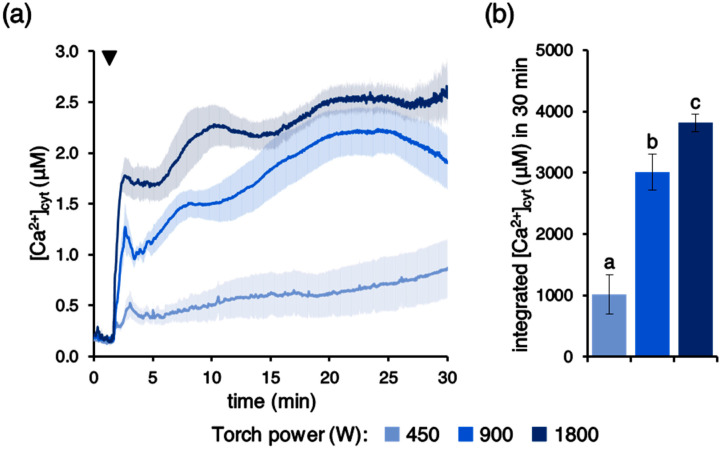
Dependence of the PAW-induced [Ca^2+^]_cyt_ elevation dynamics on the power of the plasma torch used to generate PAW. Ca^2+^ measurement assays were conducted in aequorin-expressing Arabidopsis seedlings. Seedlings were challenged with 1:2 dilutions of different PAWs, obtained after 5 min exposure of deionized H_2_O to cold plasma generated under various torch #1 power conditions: 450 W (pale blue), 900 W (blue), and 1800 W (dark blue). (**a**) Data are the means (solid lines) ± SE (shading) of six seedlings derived from three independent growth replicates. The arrowhead indicates the time of stimulus application (at 100 s); (**b**) statistical analyses of integrated [Ca^2+^]_cyt_ dynamics over 30 min. Bars labelled with different letters differ significantly (*p* < 0.05, Student’s *t* test).

We next investigated the potential dependence of PAW-induced Ca^2+^ signals on the characteristics of PAW. It is known that the production of plasma-induced chemistry and, in particular, RONS generated within the aqueous medium depends on the plasma source and on several parameters, among which is power [41]. Operating the plasma torch at different power regimes, ranging from 450 to 1800 W, was found to affect the amplitude of the recorded Ca^2+^ changes (Figure 2a,b), confirming a key role played by the modulation of the energy transferred to the pressurized gas during the generation of the cold plasma. 

**Figure 3:**
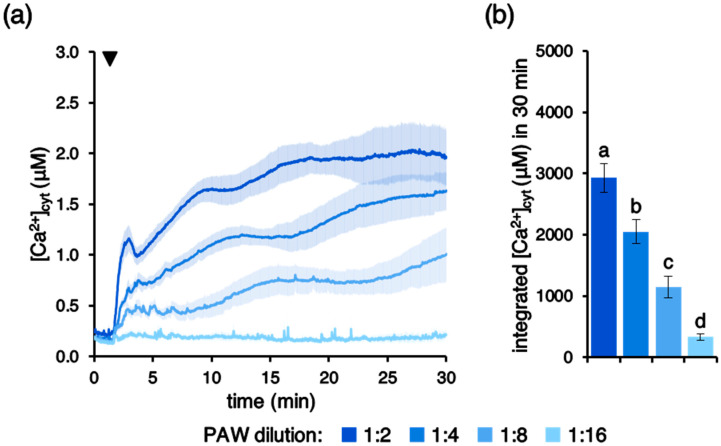
Concentration dependence of PAW-induced [Ca^2+^]_cyt_ increases. Ca^2+^ measurement assays were conducted in aequorin-expressing Arabidopsis seedlings. Seedlings were challenged with progressive dilutions of PAW (lighter colours indicate more diluted PAWs) generated by exposing deionized H_2_O to cold plasma for 5 min at 900 W torch #1 power. (**a**) Data are the means (solid lines) ± SE (shading) of six seedlings derived from three independent growth replicates. The arrowhead indicates the time of stimulus application (at 100 s); (**b**) statistical analyses of integrated [Ca^2+^]_cyt_ dynamics over 30 min are shown. Bars labelled with different letters differ significantly (*p* < 0.05, Student’s *t* test).

Ca^2+^ signals with progressively reduced magnitude were triggered by increasing ratios of PAW dilution (Figure 3a,b), demonstrating a dose-dependent effect in the PAW-induced intracellular Ca^2+^ changes triggered in Arabidopsis seedlings. 

### 2.4. Effects of Different Temperature and Time Intervals of PAW Storage on Cytosolic Ca^2+^ Changes

**Figure 4:**
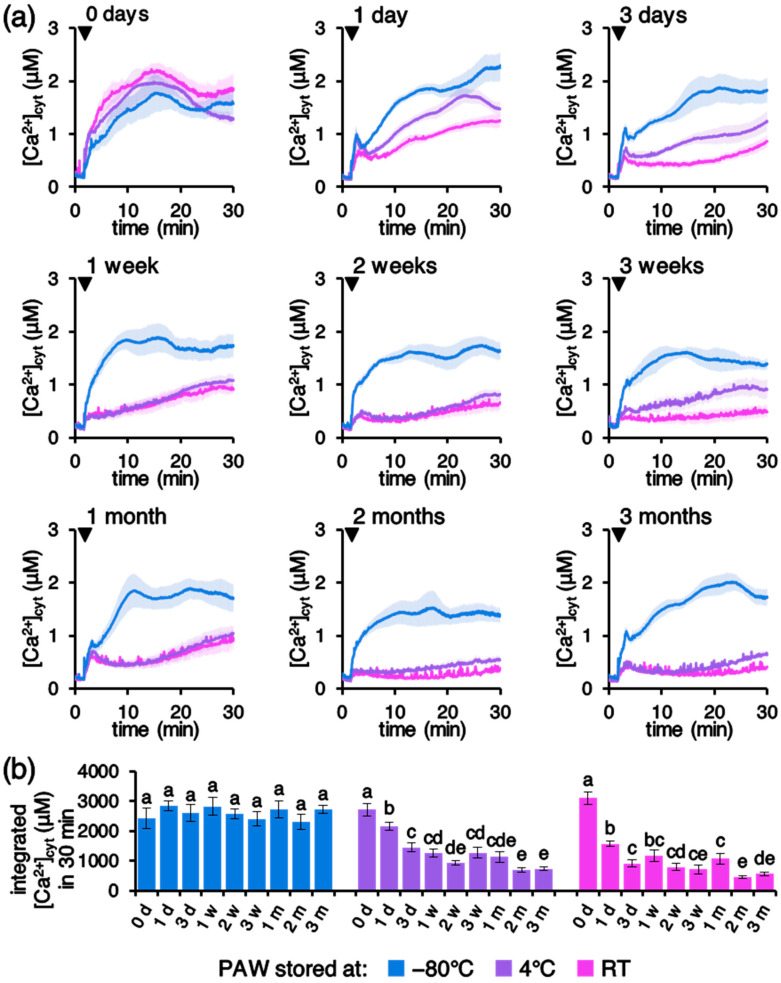
(**a**) Effects of different temperatures and time intervals of PAW storage on PAW-induced [Ca^2+^]_cyt_ increases. Ca^2+^ measurement assays were conducted in aequorin-expressing Arabidopsis seedlings. Seedlings were challenged with 1:4 dilutions of PAW generated by exposing deionized H_2_O to cold plasma for 5 min at 900 W torch #1 power. Upon production, PAW was stored at different temperatures prior to plant treatment: −80 °C (blue), 4 °C (purple), and room temperature (RT) (pink) for various time intervals (ranging from 0 days up to 3 months), as indicated on top of the panels. Data are the means (solid lines) ± SE (shading) of six seedlings derived from three independent growth replicates. Arrowheads indicate the time of stimulus application (at 100 s); (**b**) statistical analyses of integrated [Ca^2+^]_cyt_ dynamics over 30 min are shown. Key: d, days; w, weeks; and m, months. Bars labelled with different letters differ significantly (*p* < 0.05, Student’s *t* test).

An additional factor that was taken into consideration was the effect of different temperature and time intervals of PAW storage. Upon production, PAW was kept at different temperatures (−80 °C, 4 °C, and room temperature [RT]) for increasing time intervals, ranging from the immediate use up to 3 months. Figure 4 shows that PAW stored at −80 °C retains unvaried inducing activity on [Ca^2+^]_cyt_ elevations for at least 3 months. On the other hand, storage at either 4 °C or RT was found to severely affect PAW properties, resulting in greatly reduced Ca^2+^ signals already after 1 day from PAW production (Figure 4). 

### 2.5. PAW Induces a Long-Lasting Cytosolic Ca^2+^ Elevation, but Not Cell Death

**Figure 5:**
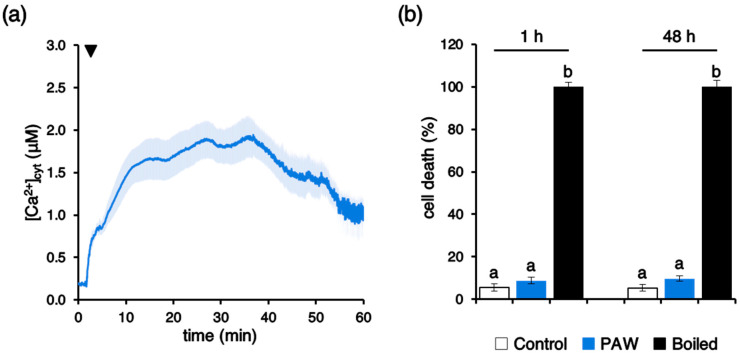
The long-lasting [Ca^2+^]_cyt_ elevation induced by PAW is not cytotoxic. (**a**) Ca^2+^ measurement assays were conducted in aequorin-expressing Arabidopsis seedlings. At 100 s (arrowhead) seedlings were challenged with a 1:4 dilution of PAW generated by exposing deionized H_2_O to cold plasma for 5 min at 900 W torch #1 power. Changes in [Ca^2+^]_cyt_ were continuously recorded for 1 h. Data are the means (solid lines) ± SE (shading) of six different seedlings derived from three independent growth replicates; (**b**) viability of Arabidopsis cell suspension cultures treated with PAW (1:4 diluted) for either 1 h or 48 h (blue bars). Control cells were incubated with cell culture medium only (white bars). The 100% value corresponds to cells treated for 10 min at 100 °C (black bars). Bars labelled with different letters differ significantly (*p* < 0.05, Student’s *t* test).

Ca^2+^ measurement assays demonstrated that the [Ca^2+^]_cyt_ elevation evoked by PAW appeared long-lasting, with sustained Ca^2+^ levels as high as ~1 µM after 1 h (Figure 5a). Nevertheless, viability assays carried out in Arabidopsis suspension-cultured cells [39] by the Evans blue test demonstrated the lack of cytotoxic effects by the PAW treatment. Indeed, no significant increase in cell death was found either at 1 h or even 48 h after PAW administration, in comparison with control samples (Figure 5b).

### 2.6. The Peculiar Chemical Environment Generated by Activation of H_2_O by Plasma Discharge Accounts for the Specific PAW-Induced Ca^2+^ Signature 

**Figure 6:**
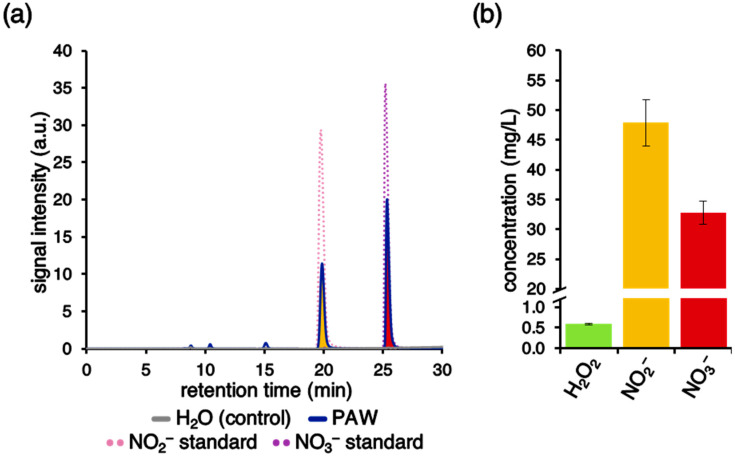
Chemical analyses for the detection of reactive oxygen and nitrogen species (RONS) in PAW. (**a**) Undiluted PAW (blue) generated by exposing deionized H_2_O to cold plasma for 5 min at 900 W torch #1 power was analysed by ion chromatography. Deionized H_2_O (grey) was used as a control. NO_2_^−^ (pink) and NO_3_^−^ (purple) solutions were used as standards. Representative traces are shown. (**b**) Determination of H_2_O_2_ (green), NO_2_^−^ (yellow), and NO_3_^−^ (red) concentrations in PAW generated in six different batches.

Chemical analyses performed by ion chromatography allowed the detection and quantification of nitrate (NO_3_^−^) and nitrite/nitrous acid (NO_2_^−^/HNO_2_) in the PAW (Figure 6a,b). The content in ammonium (NH_4_^+^), measured by ion chromatography, as well as the content of hydrogen peroxide (H_2_O_2_), measured through spectrophotometric analysis of Ti^IV^/H_2_O_2_ adduct, resulted under the detection limit of the assays [42]. Additional analyses performed by the ferrous oxidation in xylenol orange (FOX1) method [43] provided a quantification of H_2_O_2_ content at 0.59 ± 0.02 mg/L (Figure 6b).

**Figure 7:**
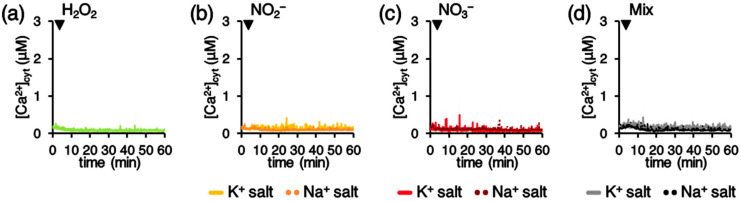
Monitoring of [Ca^2+^]_cyt_ in response to hydrogen peroxide, nitrite, and nitrate at the same doses as those measured in the PAW at 1:2 dilution. Ca^2+^ measurement assays were conducted in aequorin-expressing Arabidopsis seedlings. At 100 s (arrowheads) seedlings were challenged separately with: (**a**) 8.7 µM H_2_O_2_ (green trace); (**b**) 520.1 µM NO_2_^−^ (provided as K^+^ salt, yellow trace; provided as Na^+^ salt, orange trace); (**c**) 264.2 µM NO_3_^−^ (provided as K^+^ salt, red trace; provided as Na^+^ salt, brown trace); and (**d**) a solution (mix) containing all the above chemicals (with NO_2_^−^ and NO_3_^−^ provided as K^+^ salt, grey trace; with NO_2_^−^_and NO_3_^−^ provided as Na^+^ salt, black trace). Data are the means (solid lines) ± SE (shading) of six different seedlings derived from three independent growth replicates.

Notably, the separate administration of the single chemical components (H_2_O_2_, NO_2_^−^, and NO_3_^−^), at the same concentrations as those measured in the PAW, did not trigger detectable Ca^2+^ response in transgenic Arabidopsis seedlings (Figure 7). No differences were observed when NO_2_^−^ and NO_3_^−^ were provided as either K^+^ salts or Na^+^ salts (Figure 7). These results indicate that the unique mixture of the different chemical species found in the PAW is responsible for the induced Ca^2+^ signature.

Measurements of pH and conductivity of PAW demonstrated that the exposure of deionized H_2_O to cold plasma resulted in remarkable changes of its chemical properties. Figure S1 shows a decrease in pH from about 5.5 in deionized H_2_O to about 3.0 (in PAW generated by 5 min exposure to plasma torch #1 operating at 900 W). The extent of the pH drop was found to depend on the time interval of plasma discharge (Figure S1a), torch power (Figure S1c), and PAW dilution (Figure S1e). Likewise, the conductivity of deionized H_2_O was found to be affected by the plasma discharge, with significantly higher values in PAW obtained through increasing time intervals of exposure to plasma (Figure S1b), increasing torch powers (Figure S1d), and PAW concentration (Figure S1f). 

**Figure 8:**
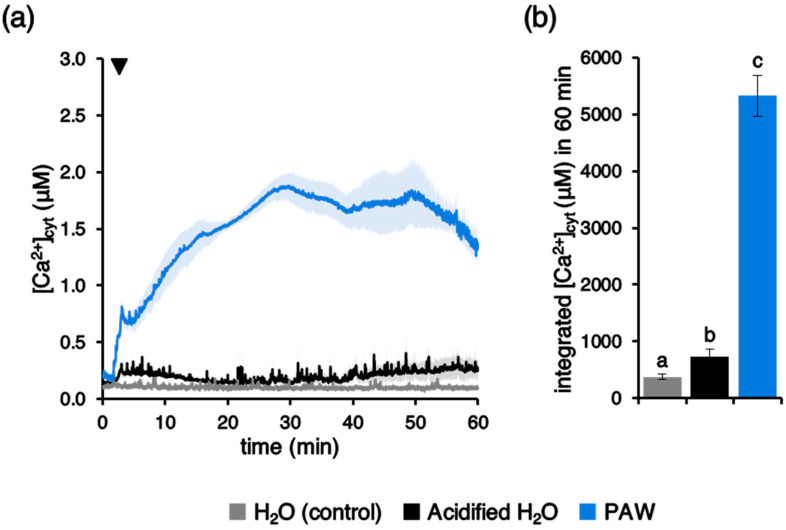
Comparison between the [Ca^2+^]_cyt_ responses induced by PAW and by an acidified H_2_O. Ca^2+^ assays were conducted in aequorin-expressing Arabidopsis seedlings. Seedlings were challenged with 1:4 dilution of PAW generated by exposing deionized H_2_O to cold plasma for 5 min at 900 W torch #1 power (blue) or deionized H_2_O acidified to the same pH as PAW at 1:4 dilution (pH 3.5) (black). Untreated deionized H_2_O (pH 5.5) was administered to control samples (grey). (**a**) Data are the means (solid lines) ± SE (shading) of six different seedlings derived from three independent growth replicates. The arrowhead indicates the time of stimulus application (at 100 s). (**b**) Statistical analyses of integrated [Ca^2+^]_cyt_ dynamics over 60 min are shown. Bars labelled with different letters differ significantly (*p* < 0.05, Student’s *t* test).

To check if the low pH could be responsible for the observed PAW-induced Ca^2+^ elevations, aequorin-expressing Arabidopsis seedlings were challenged with deionized H_2_O that had been previously acidified to the same pH as PAW at 1:4 dilution (from pH 5.5 to pH 3.5). The pH change alone was found to only slightly perturb the resting level of cytosolic Ca^2+^ (Figure 8a); therefore, the low pH-induced integrated [Ca^2+^]_cyt_ change could not account for the remarkably higher Ca^2+^ signal induced by PAW (Figure 8b). This result further confirms that the peculiar chemical environment generated in the PAW by plasma discharge is the ultimate responsible factor for the specific PAW-induced Ca^2+^ signature. In agreement with these data, no significant changes in the PAW pH were found as a consequence of PAW storage at different temperatures for different time intervals (Figure S2). 

### 2.7. The Plant Ca^2+^ Response to PAW Depends on the Total Energy Transferred to the H_2_O during Plasma Discharge 

**Figure 9:**
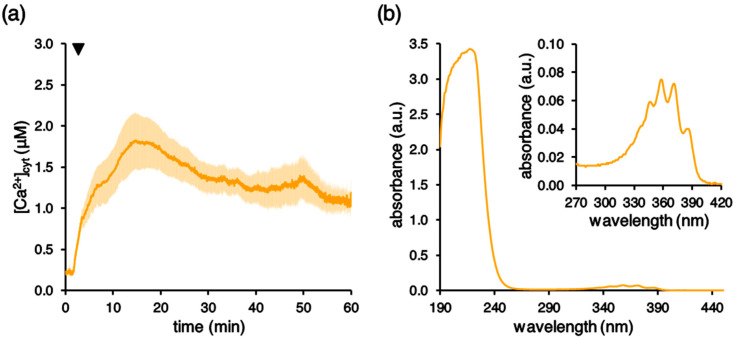
Monitoring of [Ca^2+^]_cyt_ in Arabidopsis seedlings challenged with PAW generated by an alternative plasma torch. (**a**) Ca^2+^ measurement assays were conducted in aequorin-expressing Arabidopsis seedlings. At 100 s (arrowhead) seedlings were challenged with 1:2 dilution of a PAW, obtained after 90 s exposure of deionized H_2_O to cold plasma generated by a different plasma torch (torch #2). Data are the means (solid lines) ± SE (shading) of three independent experiments. (**b**) Spectrophotometric UV-Vis analyses of the generated PAW, showing the characteristic spectrum of absorbance of nitrite, nitrate, and hydrogen peroxide. In the inset, a magnification of the region between 270 and 420 nm, highlighting the five-fingers band of NO_2_^−^ around 354 nm, is shown.

Further experiments were carried out with an additional plasma torch (torch #2) operating in a different range of power and gas pressure (see Materials and Methods). Administration of the PAW generated by this plasma source to Arabidopsis seedlings resulted in the induction of a cytosolic Ca^2+^ increase that closely mirrored the one generated by the first plasma torch (Figure 9a). Spectrophotometric analyses also confirmed, in this case, the presence of nitrate and nitrite in the PAW. Figure 9b shows the UV-Vis spectrum of the obtained PAW. The main absorption in the range 190–250 nm is ascribed to nitrite, nitrate, and hydrogen peroxide. In the 270–420 nm interval, the structured band typical of acidic NO_2_^−^ is present at 354 nm, while the maximum of the nitrate absorption reported at 300 nm is not visible due to the lower concentration of NO_3_^−^ species [44]. It must be noted that with this alternative torch, an exposure of the H_2_O to plasma lasting only 90 s was found to be sufficient to induce a Ca^2+^ increase in comparatively similar intensity to the one generated by 5 min exposure with the first torch (Figure 1). Indeed, the magnitude of the evoked plant Ca^2+^ response was found to depend on the overall power of the torch, which is the critical factor determining the energy transferred to the H_2_O during plasma discharge.

## 3. Discussion 

In the last few years there has been a surge of papers in the field of cold plasma technology. Several studies have addressed the effects of the administration of PAW to plants, concerning the promotion of growth and development [5,8,16], as well as the induction of defence responses (see [5,9,14,16] for reviews). 

In this work we focused our attention on the elucidation of the mechanisms underlying PAW perception by plants. By using an Arabidopsis line stably expressing the bioluminescent Ca^2+^ reporter aequorin in the cytosol, we demonstrated that PAW evokes rapid and sustained cytosolic Ca^2+^ elevations, characterized by specific signatures, that were found to depend upon several parameters, such as: (a) operational conditions of the torches used to generate PAW; (b) time interval of H_2_O exposure to plasma; (c) dose of PAW administered to plants; and (d) temperature and time interval of PAW storage. In particular, the magnitude of the recorded Ca^2+^ signals, measured as integral of the overall PAW-induced Ca^2+^ increases, was found to depend on the torch power, determining the energy transferred to the water during PAW generation. This was also confirmed by the use of a different torch as plasma generating device. The consistency of the results using two torches operating at different operational parameters and with different nozzle geometries has, therefore, confirmed the key role of the transferred energy to determine the Ca^2+^ signal evolution. 

The involvement of Ca^2+^ signalling in the transduction mechanisms triggered by PAW in plants could somehow be anticipated, because PAW is known to contain a mixture of ROS and RNS. In the literature, a tight link between ROS and Ca^2+^ has been firmly highlighted [45,46]. Moreover, the involvement of Ca^2+^ also in nitrate sensing is increasingly emerging [47,48]. However, it must be noted that the treatment of Arabidopsis seedlings with the same doses of H_2_O_2_, nitrate, and nitrite as those measured in the PAW did not result in detectable Ca^2+^ changes. Indeed, the concentrations of H_2_O_2_ and nitrate commonly reported as capable of inducing cytosolic Ca^2+^ elevations are much higher, i.e., in the millimolar range [24,47]. These data suggest that the induction of the PAW-induced Ca^2+^ signature may be attributable to a complex “cocktail” of different reactive chemical species contained in the PAW, rather than to a single component (such as H_2_O_2_ or nitrate). 

The PAW-induced cytosolic Ca^2+^ signature is characterized by a fast, immediate increase in [Ca^2+^]_cyt_ up to 10–25 times the resting level of the ion; notably, the Ca^2+^ change was found to reach, after about 10 min, a plateau that is maintained for additional 20 min, before slowly decreasing. Continuous monitoring of Ca^2+^ showed that even after 1 h the Ca^2+^ elevation did not dissipate completely. However, cell viability assays demonstrated the lack of cytotoxic effects of PAW in our experimental set up. An intriguing possibility is that the unique dynamics of the PAW-activated Ca^2+^ signals, i.e., a sustained, long-lasting Ca^2+^ elevation, may be a crucial determinant of the induced “priming” condition, consisting in the activation of defence gene expression and antioxidant activities that bolster plant resistance to subsequent pathogen attacks. The lack of return of [Ca^2+^]_cyt_ back to pre-stimulus resting values (about 100 nM) may lead to a state of plant alert in which the plant gets more prepared to face subsequent battles [49]. Future research will be directed at unravelling whether PAW-induced Ca^2+^ signalling underlies only plant self-defence responses or also plant growth promoting effects. 

Ca^2+^ measurement assays performed by challenging transgenic Arabidopsis seedlings with PAWs stored for different time intervals at different temperatures demonstrate that only PAW quickly frozen in liquid N_2_ upon production and then stored at −80 °C retained the ability to induce a Ca^2+^-mediated response in plants, whereas the Ca^2+^-inducing activity of PAW stored at 4 °C or room temperature rapidly decreased over time. It has previously been reported that only PAW stored at −80 °C maintained an unvaried bactericidal activity against *S. aureus*, providing important cues for optimal applications in disinfection and food preservation [50]. Our results confirm those observations about the dependence of the physico-chemical properties of PAW on temperature storage, by extending the range of PAW activities to Ca^2+^-mediated responses elicited in plants. The obtained data highlight the necessity for either cryopreservation or generation of the PAW ready-to-use and in situ, in order to allow an effective and quick administration to plants upon production. 

In summary, in this work we provided evidence that Ca^2+^ acts as an intracellular messenger in the signalling pathway triggered by PAW in Arabidopsis. Our data indicate the possibility to use aequorin-based Ca^2+^ measurements as a rapid and reliable assay to rapidly monitor early plant responses to PAW. Establishing a sound scientific ground will provide the key elements to develop tools and treatments aimed to improve plant growth and resistance to pathogens, in order to increase crop yield in a sustainable and eco-friendly way. 

## 4. Materials and Methods 

### 4.1. Plant Material and Growth Conditions

An Arabidopsis *thaliana* ecotype Columbia (Col-0) transgenic line (Cyt_YA) stably expressing in the cytosol the bioluminescent Ca^2+^ reporter aequorin fused to yellow fluorescent protein (YFP) [38,39,40] was used in this study. Seeds were surface-sterilized and sown on half-strength Murashige and Skoog medium (½ MS) (Duchefa Biochemie, Haarlem, The Netherlands) supplemented with 1.5% (*w*/*v*) sucrose, 0.8% (*w*/*v*) agar. Seedlings were grown for 7 days under a 16/8 h light/dark photoperiod at 21 °C. In some experiments, cell suspension cultures derived from the Cyt-YA line [39] were used. They were maintained and subcultured weekly in MS medium containing 0.5% (*w*/*v*) sucrose, 0.5 µg/mL 2,4-dichlorophenoxiacetic acid (2,4-D), and 0.25 µg/mL 6-benzylaminopurine (BAP) (Merck, Darmstadt, Germany), supplemented with 10 µg/mL kanamycin as selective agent, as recently described [51].

### 4.2. Generation of PAW 

PAW was generated by exposing deionized H_2_O at room temperature to the cold plasma obtained by two different plasma torches operating in a range of power 450–2700 W and with pressure from 1 to 3 bar. Torch #1 was a single rotating FLUME Jet RD1004 with an FG 1001 plasma generator (Plasmatreat, Elgin, IL, USA), that worked with an excitation frequency between 16 and 20 kHz and generated a plasma with a maximum power of 2.7 kW (Voltage = 230 V, Current = 12 A). Torch #2 was an AcXys ULS series atmospheric pressure cold plasma (AcXys Technologies, Saint-Martin-le-Vinoux, France) fed with purified air; plasma nozzle ϕ = 5 mm. Initial tests were performed varying the H_2_O amount, the distance of the nozzle from the H_2_O surface (from 1 to 10 cm), and the treatment time (from 1 to 10 min). H_2_O was kept in beakers immersed in an ice and salt cooling bath, in order to keep the H_2_O temperature increase within 40 °C for the longer treatments and the maximum power. Taking the pH as a target reference (i.e., 3.0), it has been found that the best compromise between temperature rise and pH change was, for torch #1, with the nozzle at 1.5 cm from the H_2_O surface and with treatment duration between 3 and 5 min. Concerning torch #2, the target pH was achieved with the nozzle at 10 cm from the H_2_O surface and 90 s of treatment time. Therefore, most of the results reported in this paper refer to 50 mL deionized H_2_O with its surface exposed at 1.5 cm from the torch nozzle with the standard protocol parameters set at about 900 W for 5 min concerning torch #1 and 10 cm from torch to H_2_O surface at 800 W for 90 s concerning torch #2. After generation, PAW was divided in single-use aliquots (1 mL for Ca^2+^ measurement purposes, 5 mL for pH and conductivity measurements or 25 mL for chemical analyses), immediately cryogenically frozen through immersion in liquid N_2_, and then stored at −80 °C. For long-term storage tests, some PAW aliquots were kept also at 4 °C or room temperature.

### 4.3. Chemical Analyses of PAW

Nitrite and nitrate in PAW were quantified by Dionex ICS-6000 SP ion chromatography on a Dionex IonPac ASIP-4 μm column 2 × 250 mm (Thermo Fisher Scientific, Waltham, MA, USA). Ammonium concentration was determined by Dionex Easion ion chromatography equipped with a Dionex IonPac CS12A RFIC column 4 × 250 mm (Thermo Fisher Scientific). PAW conductivity and pH were measured with electrode-based instruments, Cond7+ (XS Instruments, Carpi, Italy) and pH METER BasiC 20 (Crison, Alella, Spain), respectively.

H_2_O_2_ content in PAW was measured by spectrophotometric analysis of Ti^IV^/H_2_O_2_ adduct as described by [52]. Briefly, 0.5 mL of titanium (IV) oxysulfate solution was added to 1 mL of the sample and diluted with 8.5 mL deionized H_2_O. Spectrophotometric analysis of the peroxidic complex [Ti(O_2_)OH(H_2_O)_3_]^+^_aq_ was then carried out measuring the absorption at 409 nm with a double beam spectrometer Varian Cary 100 Bio (Varian, Palo Alto, CA, USA), using a solution containing 0.5 mL of TiOSO_4_ and 9.5 mL of deionized H_2_O as reference. H_2_O_2_ content in PAW was also determined through the FOX1 method [43]. The assay is based on a colorimetric reaction caused by the peroxide-mediated oxidation of Fe^2+^ followed by the reaction of Fe^3+^ with xylenol orange. A total of 50 μL of diluted PAW samples (consisting in 10 μL of PAW and 40 μL of H_2_O) were added to 950 μL of assay solution (0.25 mM ammonium ferrous sulfate, 25 mM H_2_SO_4_, 0.1 mM xylenol orange, and 100 mM sorbitol) and the absorbance at 560 nm was detected after 1 h incubation.

UV-Vis absorbance was measured immediately after the production of PAW by torch #2 using a double-beam UV-Vis spectrophotometer UV-2600 (Shimadzu, Kyoto, Japan) on quartz cuvettes with standard optical path of 10 mm. The spectra were recorded from 190 to 450 nm with a spectral resolution of 1 nm and a scan speed of 480 nm/min.

### 4.4. Aequorin-Based Ca^2+^ Measurement Assays

Transgenic Arabidopsis seedlings (7-day-old) were incubated overnight in the dark with 5 µM coelenterazine (Prolume, Pinetop, AZ, USA) to reconstitute the functional aequorin probe. Prior to the start of the experiment, each seedling was gently rinsed and placed in 250 µL deionized H_2_O inside the chamber of a custom-made luminometer (ET Enterprises Ltd., Uxbridge, UK), in close proximity to a low-noise photomultiplier, with a built-in amplifier discriminator. The output was captured using a photon-counting board. After 100 s, 250 µL of either PAW (tested at various dilutions) or deionized H_2_O (that has not been exposed to plasma; used as control) were injected. In some experiments, Arabidopsis seedlings were challenged with H_2_O_2_, NO_2_^−^, and NO_3_^−^ at the same concentrations as those measured in the PAW. NO_2_^−^ and NO_3_^−^ were administered as either Na^+^ or K^+^ salts (Merck). At the end of the experiment, 500 µL of a solution containing 1 M CaCl_2_, 30% (*v*/*v*) ethanol was added to completely discharge the remaining Ca^2+^ probe, allowing for the conversion of the collected light signal into [Ca^2+^]_cyt_ by means of a built-in algorithm based on the calibration curve of aequorin [53]. Integrated [Ca^2+^]_cyt_ values were obtained as the sum of each instantaneous [Ca^2+^]_cyt_ value for the entire duration of the experiment.

### 4.5. Cell Viability Assay

Cell viability was determined by the Evans blue method [54]. Briefly, Arabidopsis cell suspension cultures obtained from the transgenic line [39] were kept in control conditions or treated at mid-exponential phase (4 days) with PAW (diluted 1:4) for either 1 h or 48 h. After 15 min incubation with 0.05% (*w*/*v*) Evans blue (Merck), excess and unbound dye was removed by extensive washing with H_2_O. The dye bound to dead cells was solubilized in 1% (*w*/*v*) SDS, 50% (*v*/*v*) methanol for 30 min at 55 °C. The percentage of cell death was assessed by measuring the absorbance at 600 nm. As positive control (100% cell death), cells were incubated for 10 min at 100 °C. 

## Data Availability

The data presented in this study are available on request from the corresponding author.

## Supplementary Materials

The following are available online at https://www.mdpi.com/article/10.3390/plants10112516/s1: Figure S1, effects of different plasma exposure time intervals, torch power and PAW dilution on pH and conductivity of PAWs; Figure S2, effect of time interval and temperature of PAW storage on pH.
