# Supplementary material for: Plasma-Activated Water Triggers Rapid and Sustained Cytosolic Ca2+ Elevations in Arabidopsis thaliana
Source: Plants (Basel). 2021 Nov 19;10(11):2516. doi: 10.3390/plants10112516 (PMC8622995; doi:10.3390/plants10112516)
Supplement: Supplementary file 1 [file plants-10-02516-s001.zip › plants-1462880-supplementary.pdf]

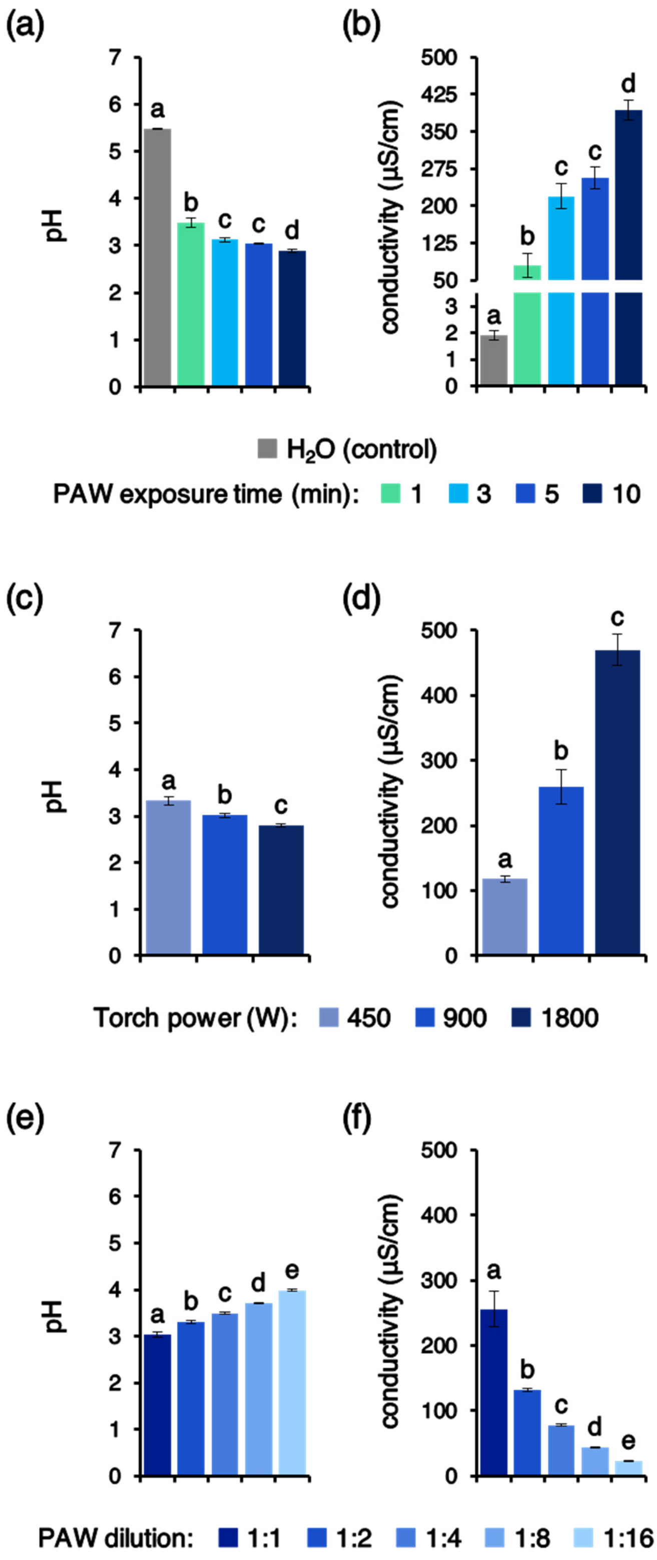

Figure S1. Effects of different plasma exposure time intervals, torch powers and PAW dilutions on pH and conductivity of PAWs. (**a-b**) pH and conductivity were measured in untreated deionized H_2O (grey, control) and different undiluted PAWs, obtained by exposing deionized H_2O to cold plasma, generated at 900 W torch #1 power, for 1 min (green), 3 min (light blue), 5 min (blue) or 10 min (dark blue), respectively; (**c-d**) pH and conductivity were measured in different undiluted PAWs, obtained by 5 min exposure of deionized H_2O to the cold plasma generated under various torch #1 power conditions: 450 W (pale blue), 900 W (blue); 1800 W (dark blue); (**e-f**) pH and conductivity were measured in progressive dilutions of PAW (lighter colours indicate more diluted PAWs, as indicated under the panels) generated by exposing deionized H_2O to cold plasma for 5 min at 900 W torch #1 power. Data are the means ± SE of the values obtained in PAW generated in 3 different batches. Bars labelled with different letters differ significantly ($P < 0.05$, Student's *t* test).

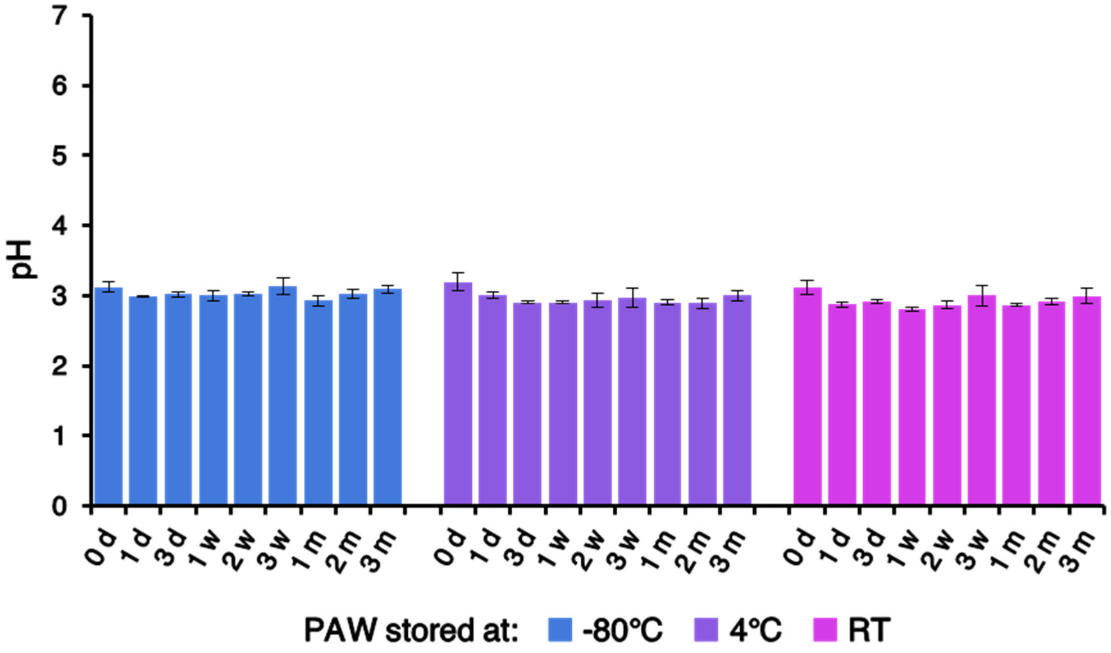

Figure S2. Effects of different time intervals and temperatures of PAW storage on pH. PAW was generated by exposing deionized H_2O to cold plasma for 5 min at 900 W torch #1 power. pH of PAW was measured after storage at different temperatures (-80°C, blue; 4°C, purple; room temperature (RT), pink) for various time intervals (ranging from 0 days up to 3 months). Data are the means ± SE of two different PAW preparations, giving very similar results. No statistical differences were observed (Student's *t* test). d, days; w, weeks; m, months.
